# NPCARE: database of natural products and fractional extracts for cancer regulation

**DOI:** 10.1186/s13321-016-0188-5

**Published:** 2017-01-05

**Authors:** Hwanho Choi, Sun Young Cho, Ho Jeong Pak, Youngsoo Kim, Jung-yun Choi, Yoon Jae Lee, Byung Hee Gong, Yeon Seok Kang, Taehoon Han, Geunbae Choi, Yeeun Cho, Soomin Lee, Dekwoo Ryoo, Hwangseo Park

**Affiliations:** 1 Department of Bioscience and Biotechnology, Institute of Anticancer Medicine Development, Sejong University, 209 Neungdong-ro, Kwangjin-gu, Seoul, 05006 Korea; 2 College of Korean Medicine, Dongguk University, 27 Dongguk-ro, Ilsandong-gu, Goyang-si, Gyeonggi-do 10326 Korea; 3 Department of Obstetrics and Gynecology, College of Medicine, The Catholic University of Korea, 222 Banpo-daero, Sucho-gu, Seoul, 06591 Korea; 4 Department of Biological Sciences, KAIST, 291 Daehak-ro, Yuseong-gu, Daejeon, 34141 Korea; 5 Graduate School of Medical Science and Engineering, KAIST, 291 Daehak-ro, Yuseong-gu, Daejeon, 34141 Korea; 6 Department of Korean Gynecology, Jaseng Hospital of Korean Medicine, 858 Eonju-ro, Gangnam-gu, Seoul, 06017 Korea; 7 College of Korean Medicine, Wonkwang University, 460 Iksandae-ro, Iksan, Jeonbuk 54538 Korea; 8 WiFun Team, Nexon, 7 Pangyo-ro 256 gil, Bundang-gu, Seongnam-si, Gyeonggi-do 13487 Korea; 9 Hanvit Dental-Medical Hospital, 396 Seowon-daero, Wonju, Gangwon-do 26484 Korea; 10 Department of Clinical Korean Medicine, Kyung Hee University Korean Medicine Hospital, 26 Kyungheedae-ro, Dongdaemun-gu, Seoul, 02447 Korea

**Keywords:** NPCARE, Natural product, Fractional extract, Anticancer medicine, Database

## Abstract

**Background:**

Natural products have increasingly attracted much attention as a valuable resource for the development of anticancer medicines due to the structural novelty and good bioavailability. This necessitates a comprehensive database for the natural products and the fractional extracts whose anticancer activities have been verified.

**Description:**

NPCARE (http://silver.sejong.ac.kr/npcare) is a publicly accessible online database of natural products and fractional extracts for cancer regulation. At NPCARE, one can explore 6578 natural compounds and 2566 fractional extracts isolated from 1952 distinct biological species including plants, marine organisms, fungi, and bacteria whose anticancer activities were validated with 1107 cell lines for 34 cancer types. Each entry in NPCARE is annotated with the cancer type, genus and species names of the biological resource, the cell line used for demonstrating the anticancer activity, PubChem ID, and a wealth of information about the target gene or protein. Besides the augmentation of plant entries up to 743 genus and 197 families, NPCARE is further enriched with the natural products and the fractional extracts of diverse non-traditional biological resources.

**Conclusions:**

NPCARE is anticipated to serve as a dominant gateway for the discovery of new anticancer medicines due to the inclusion of a large number of the fractional extracts as well as the natural compounds isolated from a variety of biological resources.

**Electronic supplementary material:**

The online version of this article (doi:10.1186/s13321-016-0188-5) contains supplementary material, which is available to authorized users.

## Background

Although a great deal of efforts has been devoted to the development of therapeutics for a long time, cancer represents the major reasons for human death at an increasing pace. Because the discovery of anticancer medicine lags behind the rapid increase in the pathogenesis of cancer, more than 10 million people are expected to die of cancer in 2020, which corresponds to approximately 20% of all human deaths. The difficulty in the development of anticancer medicines is well reflected in the fact that only 5% of the candidates entering clinical trials reach the approval for marketing [1]. To promote the discovery of anticancer medicines, it is necessary to enrich the chemical and biological resources from which one can select a promising molecular scaffold as the starting point of the development.

With respect to the lead discovery, it is worth noting that natural products and their direct derivatives occupy 34% of new drugs approved over a few decades by US Food and Drug Administration (FDA) [2]. Besides the possession of unique pharmacophores and a high degree of stereochemistry, natural products are superior to the synthetic compounds in terms of the delivery to the intracellular site of action because most of them belong to the biologically active metabolites that should be the actual substrates of membrane transport systems [3]. Furthermore, natural products tend to have the better bioavailability than the synthetic compounds, which prevents them from being the false positives in the early stage of discovery [4].

Accordingly, several online databases for natural products have been constructed to provide a systematic and versatile platform for drug discovery including SuperNatural [5], CancerResource [6], NPACT [7], TCMSP [8], CancerHSP [9], TCMID [10], and Phytochemica [11]. In addition to three dimensional structures of commercially available natural products and the interactions with the target proteins, these databases contain the pharmacological properties associated with absorption, distribution, metabolism, excretion, and toxicity (ADMET) as well as in vitro and in vivo anticancer activities. Despite the prevalence of publicly available databases, the number of the collected natural products with anticancer activity ranges from 1000 to 4000, which would be insufficient to serve as a breakthrough chemical library for lead generation. Furthermore, information is missing or very limited about the extract mixtures in the existing natural product databases although the traditional Chinese medicines have been very useful for finding the promising leads with respect to various pharmacological targets [12–14].

To provide information for a sufficient number of the natural products and the extract mixtures with anticancer activity to research communities worldwide through Open Access protocol, we construct an online database referred to as Natural Products for Cancer Regulation (NPCARE, http://silver.sejong.ac.kr/npcare). More specifically, NPCARE aims to complement and augment the public data repositories by the enrichment of the natural products and the fractional extracts isolated not only from plants but also from diverse non-traditional biological resources including marine organisms, fungi, and bacteria. NPCARE is therefore likely to serve as a comprehensive public resource from which users can select a good starting point for the discovery of anticancer medicines.

## Data collection and assembly

**Fig. 1:**
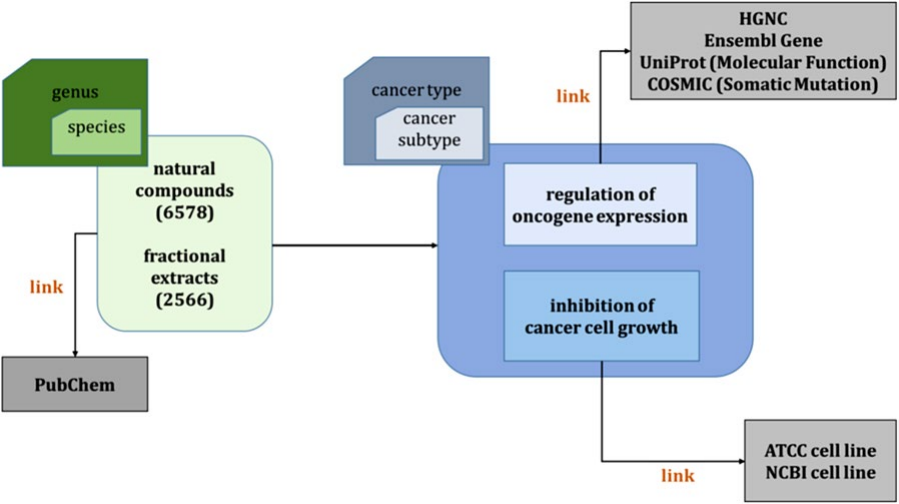
Schematic depiction of the overall strategy for constructing the NPCARE database

The overall strategy for constructing the NPCARE database is depicted in Fig. 1. To obtain an extensive repertoire of natural products and extract mixtures, numerous biological data had to be manually compiled from the literature and web resources. This began with the searches of PubMed (http://www.ncbi.nlm.nih.gov/pubmed) with genus and species names of plants, marine organisms, fungi, and bacteria that contained the natural products and/or the extract mixtures with anticancer activity. A total of approximately 20,000 articles were retrieved from 150 scientific journals pertaining to natural products, cancer, and medicinal chemistry. The journals that made a significant contribution to constructing the NPCARE database include *Journal of Natural Product*, *Phytomedicine*, *Planta Medica*, *Natural Product Reports*, *Journal of Ethnopharmacology*, *Cancer Research*, *Anticancer Research*, *British Journal of Cancer*, *Carcinogenesis*, *Cancer Letters*, and *Biological and Pharmaceutical Bulletin*. All the retrieved scientific papers were inspected to obtain and catalogue the major contents of database such as compound names, extracts, cell lines used for cytotoxicity assays, cancer types, and target gene or protein. With respect to the data mining, multiple genus and species names were unified according to the taxonomy database provided by NCBI (https://www.ncbi.nlm.nih.gov/taxonomy). We referred to PubChem and the original articles for nomenclature and stereochemistry of all the compounds in NPCARE. The data collected from all the resources were then merged to construct a non-redundant library containing a total of 6578 unique natural products and 2566 fractional extracts. Although the vast majority of natural products and extracts originated from eudicots and monocots, good anticancer agents could also be found from some marine organisms such as sponges and corals as well as from ascomycetes, basidiomycota, and bacteria. The list of all genus and species names is provided in Additional file 1.

Whenever the natural product or the extract mixture was found in the resources, we monitored the cell line used for validating the anticancer activity with respect to the cancer types classified by international classification of disease (ICD) of version 10. For each cancer type, the subtypes determined by histological classifications were also specified as exemplified by squamous cell lung cancer, big cell lung cancer, and adenocarcinoma for lung cancer. Two criteria were applied separately to confirm the presence of anticancer activity. First, the natural products and the fractional extracts were adopted as the element of NPCARE if they could inhibit the growth of cancer cell line. Each cell line used to assess the anticancer activity was hyperlinked to American type culture collection (ATCC) or National Center for Biotechnology Information (NCBI) for further information. The anticancer activity of a natural product and fractional extract was also assessed by the capability to downregulate the expressions of oncogenes or to upregulate the cancer-suppressing genes, which were implicated with the change in mRNA transcription levels. In this case, the gene names were also hyperlinked to the web pages of HUGO gene nomenclature committee (HGNC), Ensembl genome browser, UniProt knowledgebase, and catalogue of somatic mutations in cancer (COSMIC) so that users can refer to the details of the target gene. NPCARE also offers the hyperlink to PubChem for further information about the physicochemical and pharmacological properties of the natural compounds whose anticancer activities were verified.

## Contents and data retrieval

Both natural products and fractional extracts reveal a wide-spectrum distribution with respect to the biological resources and the cancer types. The NPCARE information table for a natural product includes the caner type, the scientific names of the resource organism, the compound name, PubChem ID, the names of the target gene or protein, UniProt ID of the target gene, the biological function of the target protein, COSMIC and Ensembl ID’s of the target gene, the pattern for the target gene regulation, and the cell line used for measuring the anticancer activity. The same is true of a total of 2566 extract mixtures. For each entry of NPCARE, also hyperlinked is the original scientific paper or the open resource at which the anticancer activity was reported. Users can download the entire database file by simply clicking “NPCARE CSV Download” tab in the “Useful Links” menu at NPCARE website.

**Fig. 2:**
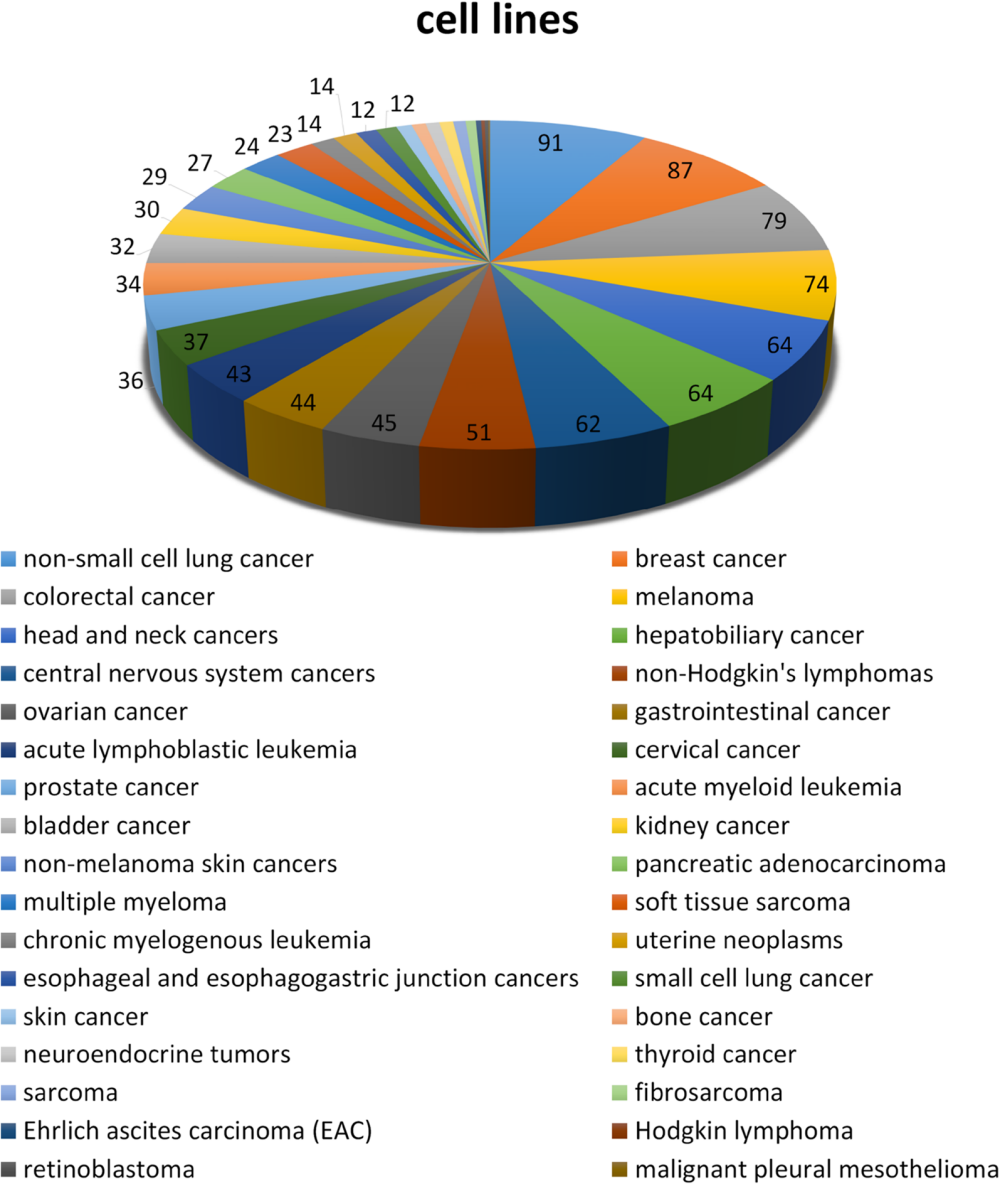
Number distribution of the cell lines in NPCARE with respect to various cancer types and subtypes

At present, NPCARE covers 532 cell lines for 34 cancer types. Figure 2 illustrates the distribution of the cell lines with respect to the cancer types. More than 70 kinds of cell lines are collected in NPCARE for popular and lethal cancers such as non-small cell lung cancer, breast and colorectal cancers, and melanoma while only a few are available for rare cancers including Ehrlich ascites carcinoma, Hodgkin lymphoma, and retinoblastoma. For a specific cancer type, users can retrieve the list of natural products and fractional extracts whose anticancer activities were validated with varying cancer cell lines.

**Fig. 3:**
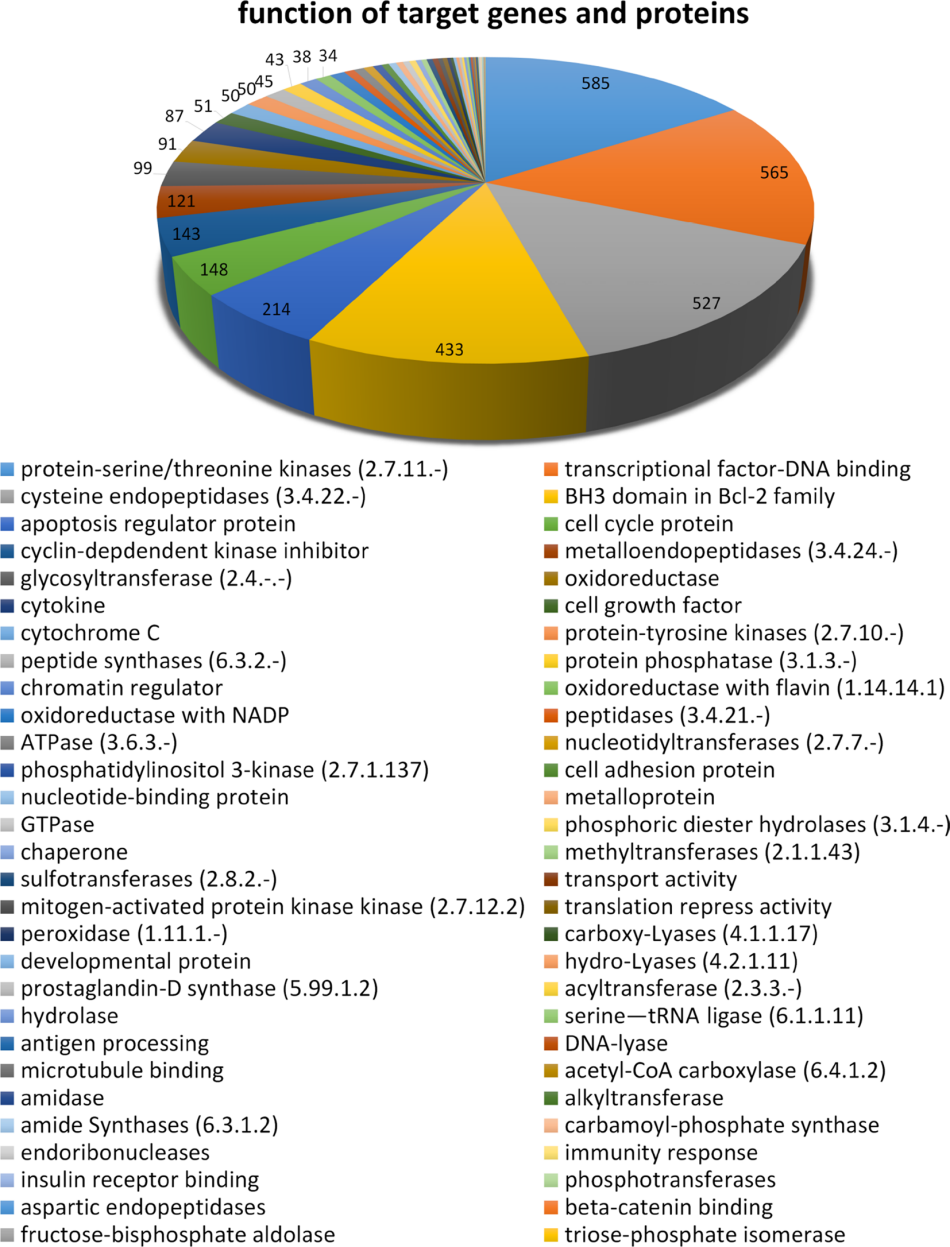
Distribution of the natural products in NPCARE with respect to the target proteins responsible for the pathogenesis of cancer

As can be seen in Fig. 3, the natural products in NPCARE are distributed among the diverse molecular targets including 58 classes of proteins that have served as the receptor models in the development of anticancer medicines. In particular, more than 500 natural products appear to be capable of modulating the activities of kinases, transcription factors, and cysteine caspases, which are responsible for the pathogenesis of human cancers by affecting the cellular signal transduction, the expression of oncogenes, and apoptosis, respectively. A large number of natural anticancer compounds in NPCARE are also associated with BH3 domain of Bcl-2 family, various proteins regulating the cell cycle and apoptosis, and cyclin-dependent kinase inhibitors. It is also worth noting that NPCARE contains 171 natural products acting on metalloproteins including metallopeptidases. This would be the merit of NPCARE in the context that it is difficult to design a molecule that can bind tightly to metalloproteins due to the difficulty in finding a suitable chemical moiety to coordinate the central metal ion cofactor [15]. Furthermore, most metal-binding groups have poor physicochemical properties as a drug candidate because they have inevitably to contain a highly polar moiety to coordinate the positively charged metal ion. The natural products that modulate the expression of a metalloprotein target can therefore be good alternative for the drug candidates binding in its active site.

**Fig. 4:**
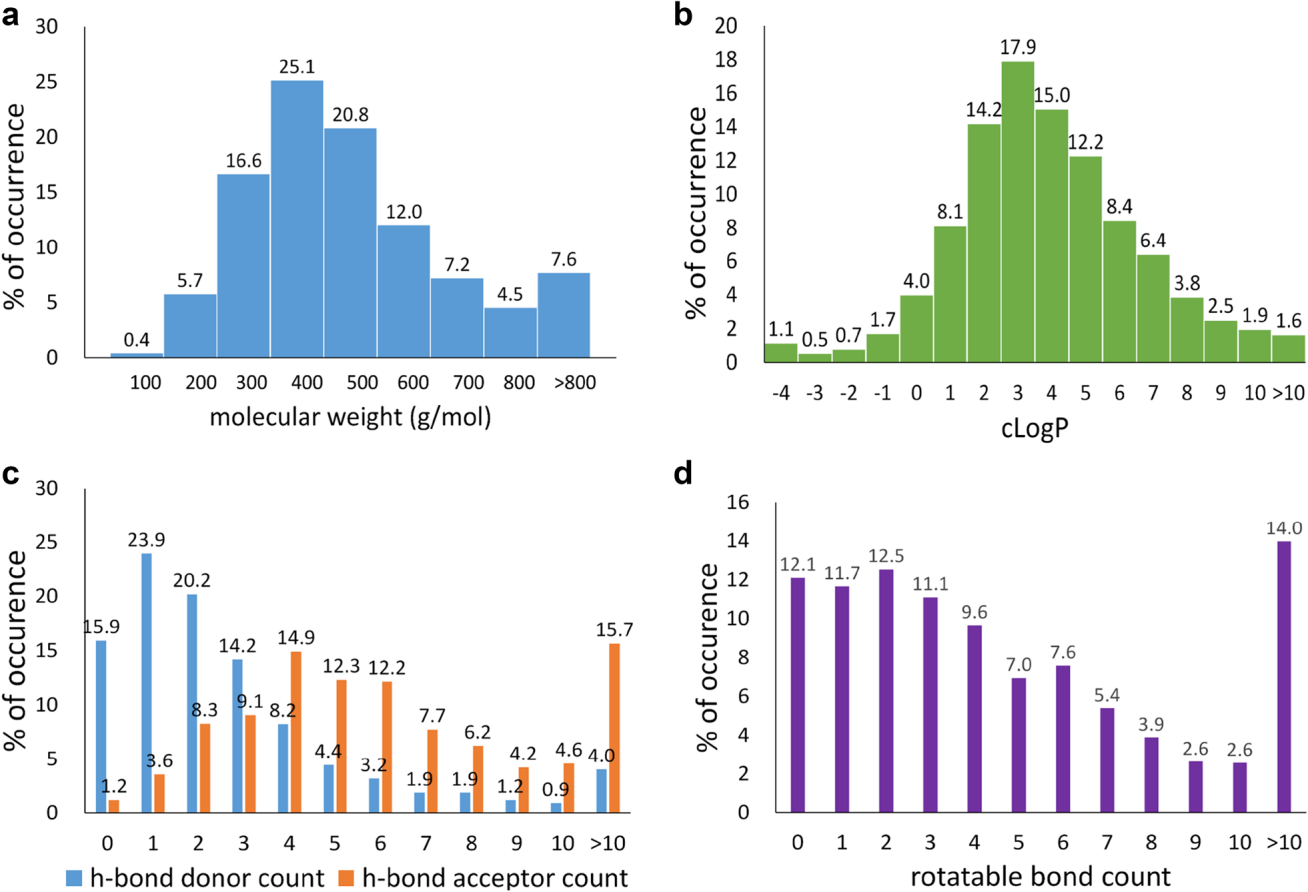
Distributions of **a** molecular weight, **b** cLogP, **c** numbers of hydrogen bond donors and acceptors, and **d** number of rotatable bonds in the natural products of NPCARE

To evaluate the natural products in NPCARE as a potential drug candidate, we analyzed their physicochemical properties associated with ADMET: molecular weight (MW), calculated partition coefficient (cLogP), numbers of hydrogen bond acceptors (HBA) and donors (HBD), and number of rotatable bonds. As shown in Fig. 4, MWs of the natural products in NPCARE follows a Poisson-like distribution and peak in the range of 300–400 amu whereas the statistics of cLogP values exhibit a Gaussian-like distribution at the maximum around 2.5. The majority of natural products in NPCARE have HBD and HBA atoms in the ranges of 0–5 and 2–8, respectively, as similar to the drug molecules in the market. Because the number of rotatable bonds falls within 10 in the majority of NPCARE compounds (Fig. 4d), they would be capable of binding to the biomolecular targets without a significant loss of entropy. Overall, 57.4% of the natural products in NPCARE satisfy all the Lipinski’s rules for drug-likeness [16].

Judging from the good physicochemical properties, NPCARE molecules are expected to enrich the chemical library from which a good lead compound for anticancer medicine can be identified with virtual screening. Because the natural products account for a large portion of the approved drugs [17], they have often served as the structural core to optimize the pharmacological activity within a series of similar compounds. Future updates of NPCARE will be focused on the increase in the number of natural products and simultaneously on the addition of the force field parameters for each natural product to facilitate the virtual screening of the potential inhibitors of various target proteins.

**Fig. 5:**
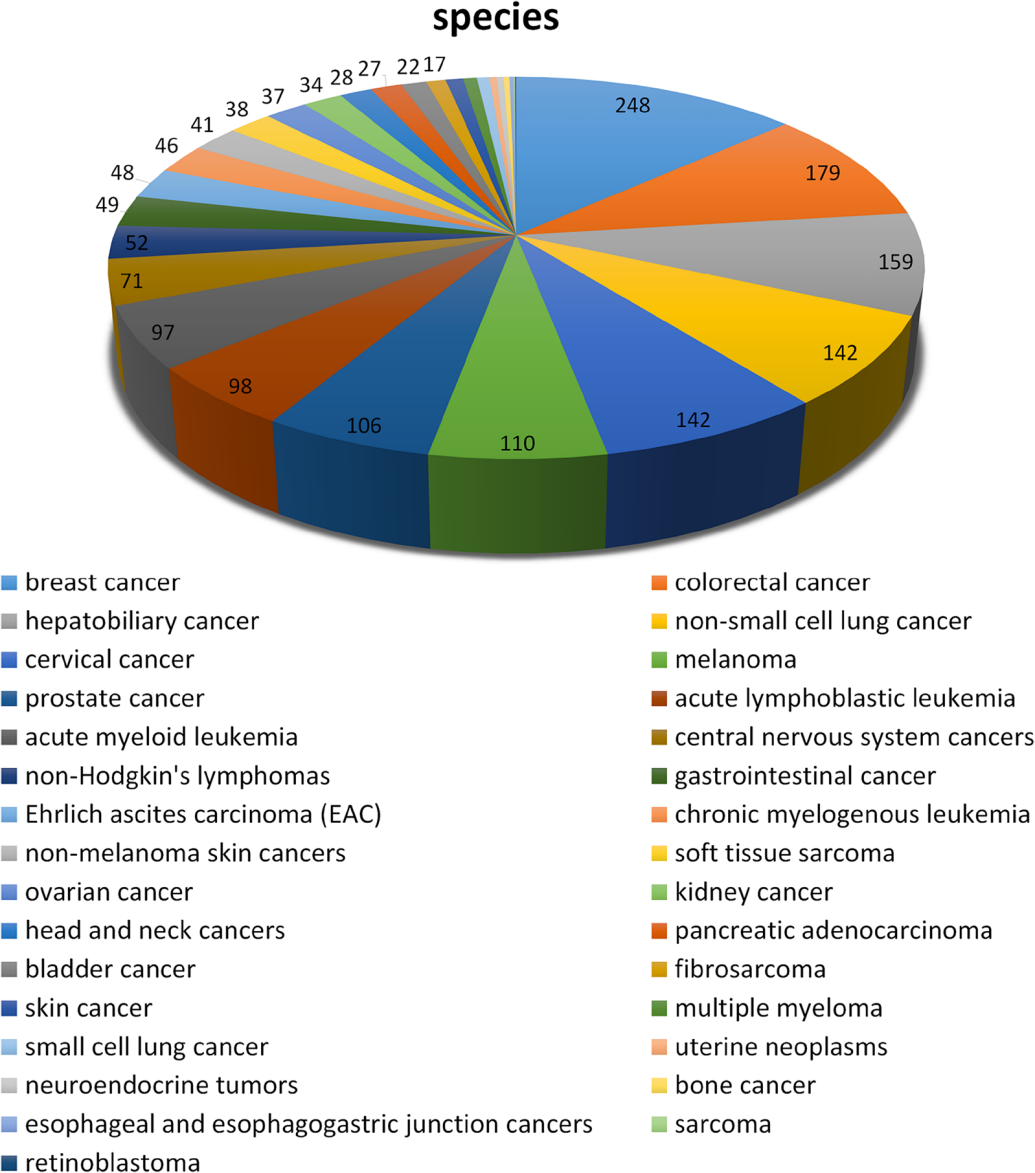
Number distribution of the biological species to produce the fractional extracts with the anticancer activity for individual cancer types

Also collected in NPCARE are 2566 fractional mixtures isolated from 1952 distinct species including plants, marine organisms, fungi, and bacteria. Now the fractional extracts with anticancer activity are available for 31 cancer types. Figure 5 shows the number of biological species producing the fractional extracts with anticancer activity. This number distribution appears to be similar to that of the cancer cell lines among the cancer types (Fig. 2) in that the occurrences are high and low for prevalent and rare cancers, respectively. In order to show the potential ingredients of the extract mixture of interest, NPCARE allows for users to retrieve the list of the natural products contained in each resource organism according to its genus and species names.

**Fig. 6:**
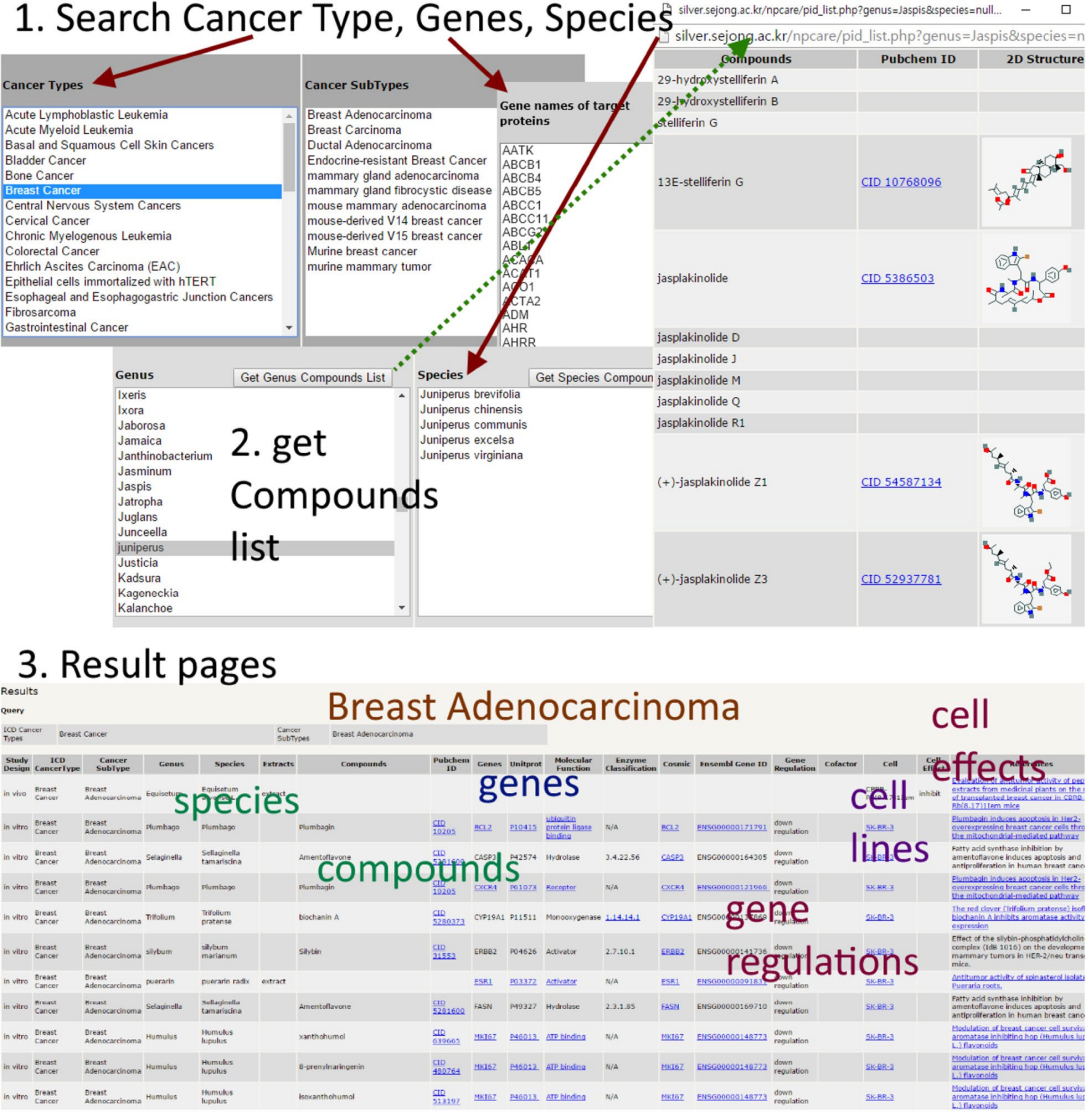
Schematic workflow of NPCARE for the input query with the cancer type, the target gene or protein, and the taxonomic names of the resource organism

The data in NPCARE can be readily accessed using the several options. As shown in Fig. 6, users can search the natural anticancer compounds and extracts according to the cancer type, the target gene or protein, and the taxonomic names of the resource organism. The search results will appear at the end of web page in the tabulated form. Each entry contains the compound name, PubChem ID, detailed information about the target gene or protein, and the cell line used to measure the anticancer activity. Some data are hyperlinked to various external databases and web resources including PubChem, UniProtKB, ExPASy, HGNC, COSMIC, Ensembl, ATCC, and NCBI at which further information is available for the natural product of interest, the target gene or protein, and the cell line.

The result table lists not only the natural products but also the fractional extracts. The potential components of each fractional extract are displayed by clicking on ‘Get Genus Compounds List’ or ‘Get Species Compounds List’ tabs after selecting the genus and/or species names of the resource organism. All or some of the ingredients in the extract mixture may be responsible for the anticancer activity in the additive or synergistic fashions. Because the extract mixtures usually contain a number of the unknown secondary metabolites, they may offer a good opportunity to identify the novel candidate for anticancer medicine as exemplified in the discovery of chlorofusin by screening the microbial extracts to find the inhibitors of the p53–MDM2 interaction [18]. The unique structural scaffolds of natural products can make it possible to construct a chemical library that retains the highly relevant three-dimensional aspects in cyclization and chirality. These structural peculiarities have the advantage of offering the selectivity in binding to the biomolecular target and thereby reducing the potential side effects.

Most drugs of natural origin in the clinical market stem from the plants with approximately 60% of them being clustered into only 10 taxonomic families [19]. However, the number of plant families is extended to 197 (743 types of genus) in NPCARE, which would be beneficial to users who are seeking the new candidates for the natural anticancer medicine. Furthermore, the contents of NPCARE are augmented with the natural products and the fractional extracts isolated from the non-traditional biological resources including 164, 120, and 48 species of marine organisms, fungi, and bacteria, respectively. The inclusion of such non-traditional resources seems to be necessary because they proved to contain a variety of natural products that developed into a new anticancer medicine [20, 21]. Judging from the enrichment of the natural products and the fractional extracts isolated from a variety of plants and non-traditional biological resources, NPCARE is anticipated to serve as a useful platform for the discovery of the new natural anticancer medicines.

## Conclusion

The construction of NPCARE database aims to promote the development of anticancer medicines from the natural resources. Each natural product entry seems to serve as a molecular scaffold using which the anticancer activity can be optimized by quantitative structure–activity relationship analysis. Users would also be able to identify the new starting points for the development of natural anticancer medicine by exploring the potential components of the fractional extracts with anticancer activity. Besides the augmentation of the traditional plant resources, NPCARE is further enriched with the natural products and the fractional extracts isolated from a variety of non-traditional biological resources. As a consequence, NPCARE contains 6578 natural compounds and 2566 fractional extracts isolated from 1952 biological resources including plants, marine organisms, fungi, and bacteria whose anticancer activities have been validated with 1107 cell lines for 34 cancer types. It is also allowed for users to construct a chemical library of the natural products specific for a cancer type as well as for a target gene or protein. Judging from the considerable amount of entries with respect to diverse resource organisms, NPCARE is anticipated to serve as a comprehensive computational platform for the discovery of new anticancer medicines.

## Additional file

**Additional file 1.** The list of genus and species names contained in NPCARE.

## Abbreviations

NPCARE: natural products and fractional extracts for cancer regulation
FDA: Food and Drug Administration
ADMET: absorption, distribution, metabolism, excretion, and toxicity
ICD: international classification of disease
ATCC: American type culture collection
NCBI: National Center for Biotechnology Information
HGNC: HUGO gene nomenclature committee
COSMIC: catalogue of somatic mutations in cancer
MW: molecular weight
cLogP: calculated partition coefficient
HBA: hydrogen bond acceptor
HBD: hydrogen bond donor
