# Supplementary material for: NPCARE: database of natural products and fractional extracts for cancer regulation
Source: J Cheminform. 2017 Jan 5;9:2. doi: 10.1186/s13321-016-0188-5 (PMC5267755; doi:10.1186/s13321-016-0188-5)
Supplement: Supplementary file 1 — Additional file 1. The list of genus and species names contained in NPCARE. [file 13321_2016_188_MOESM1_ESM.doc]

**List of genus and species names contained in NPCARE**

Aaptos aaptos

Aaptos suberitoides

Albatrellus confluens

Abelmoschus esculentus

Abelmoschus moschatus

Abies alba

Abrus precatorius

Acacia burkittii

Acacia confusa

Acacia farnesiana

Acacia mellifera

Acacia nilotica

Acacia pennata

Acacia salicina

Acacia victoriae

Acalypha alopecuroidea

Acanthella cavernosa

Acanthopanax koreanum

Acanthopanax senticosus

Acanthospermum australe

Acanthospermum hispidum

Acanthus ilicifolius

Acer mandshuricum

Acer maximowiczianum

Acer tegmentosum

Acetobacter aceti

Acetobacter xylinum

Achyranthes aspera

Achyranthes bidentata

Achyrocline satureioides

Acinetobacter baumannii

Aconitum carmichaeli

Acorus calamus

Acorus gramineus

Acremonium exuviarum

Acroptilon repens

Actinia equina

Actinidia chinensis

Actinidia eriantha

Actinidia polygama

Actinidia valvata

Actinomadura atramentaria

Actinoplanes utahensis

Adenia cissampeloides

Adenia gummifera

Adenium obesum

Aeginetia indica

Aegle marmelos

Artemisia princeps

Aeromonas

Aeromonas salmonicida

Aerva lanata

Aesculus hippocastanum

Agaricus bisporus

Agaricus blazei

Agaricus brasiliensis

Agaricus sylvaticus

Agave sisalana Perrine

Agelas nakamurai

Ageratum conyzoides

Aglaia crassinervia

Aglaia edulis

Aglaia elliptica

Aglaia elliptifolia

Aglaia foveolata

Aglaia odorata

Aglaia oligophylla

Aglaia rubiginosa

Aglaia silvestris

Aglaia spectabilis

Agrimonia pilosa

Agrobacterium

Ailanthus excelsa

Ajuga decumbens

Akebia quinata

Alangium longiflorum

Albizia amara

Albizia gummifera

Albizia julibrissin

Alectoria ochroleuca

Alertigorgia

Ailanthus altissima

Alisma canaliculatum

Allium cepa

Allium sativum

Allium tuberosum

Alnus glutinosa

Alnus hirsuta

Alnus japonica

Alnus sieboldiana

Aloe arborescens

Aloe ferox

Aloe maculata

Aloe

Aloe vera

Alpinia galanga

Alstonia angustiloba

Alstonia macrophylla

Alstonia scholaris

Alternanthera sessilis

Alternaria alternata

Alternaria porri

Alternaria sonchi

Alternaria sp. ZJ9-6B

Alternaria tenuis

Amaranthus gangeticus

Amaranthus tricolor

Amaranthus viridis

Amentotaxus formosana

Amomum aculeatum

Amomum subulatum

Amomum tsao-ko

Amorphophallus konjac

Ampelomyces quisqualis

Ampelopsis cantoniensis

Amphidinium

Amphimedon

Amsinckia

Amycolatopsis

Amyris elemifera

Anabaena laxa

Anabaena sp. PCC 7120

Anabaena torulosa

Anacardium occidentale

Anadenanthera colubrina

Ananas comosus

Andrographis paniculata

Androsace integra

Androsace umbellata

Anemarrhena asphodeloides

Angelica gigas

Angelica purpuraefolia

Angelica sinensis

Aniba rosaeodora

Anisomeles indica

Annona cherimola

Annona diversifolia

Annona glabra

Annona hypoglauca

Annona montana

Annona muricata

Annona reticulata

Annona senegalensis

Annona squamosa

anthemis maritima

Anthemis ruthenica

Cota segetalis

Antipathes dichotoma

Antrodia camphorata

Antrodia camphorate

Aphanamixis grandifolia

Aphanamixis polystachya

Apiospora montagnei

Aplidium

Aplysia dactylomela

Aplysia punctata

Aplysina aerophoba

Aplysina caissara

Aplysinopsis

Aralia cordata thunb.

Aralia continentalis

Aralia decaisneana

Aralia elata

Arbutus unedo

Arca granosa

Archangium gephyra

Arctium lappa

Arctostaphylos uva-ursi

Ardisia compressa

Ardisia crenata

Ardisia japonica

Areca catechu

Arenicola cristata

Arisaema amurense

Arisaema decipiens Schott

Arisaema flavum

Arisaema jacquemontii Blume

Arisaema tortuosum

Arisarum vulgare

Aristolochia baetica

Aristolochia heterophylla

Aristolochia macroura

Aristolochia mollissima

Aristolochia triangularis

Arnica montana

Artemisia absinthium

Artemisia annua

Artemisia argyi

Artemisia capillaris

Artemisia diffusa

Artemisia iwayomogi

Artemisia vestita

Arthrinium

Arthrobacter

Artocarpus altilis

Artocarpus communis

Artocarpus elasticus

Artocarpus heterophyllus

Artocarpus kemando

Artocarpus lakoocha

Artocarpus lanceifolius

Artocarpus nitidus subsp. lingnanensis

Asarum heterotropoides

Aspalathus linearis

Asparagus adscendens

Asparagus racemosus

Aspergillus fumigatus

Aspergillus niger

Aster tataricus

Astragalus membranaceus

Atractylodes lancea

Avena sativa

Axinella carteri

Axinella polypoides

Axinyssa ambrosia

Bacillus anthracis

Baileya multiradiata

Barleria prionitis

Barringtonia racemosa

Botrytis bassiana

Bauhinia variegata

Begonia nantoensis

Berberis amurensis

Berberis aristata

Beta vulgaris

Betula platyphylla

Bidens alba

Actaea racemosa

Blechnum orientale

Bletilla striata

Blumea balsamifera

Boehmeria japonica

Boehmeria pannosa

Boerhaavia diffusa

Boesenbergia rotunda

Bolbostemma paniculatum

Boswellia serrata

Bowdichia nitida

Brachylaena ramiflora

Brassica oleracea

Brassica campestris

Breynia fruticosa

Briareum excavatum

Briareum

Bromelia fastuosa

Brucea Javanica

Bryonia

Bryopsis

Bufo gargarizans

Bugula neritina

Bulbophyllum odoratissimum

Bupleurum Kaoi

Bupleurum scorzonerifolium

Bursatella leachii

Butea Monosperma

Butea superba

Buxus microphylla

Buxus sempervirens

Byssochlamys nivea

Cachrys

Cacospongia mycofijiensis

Caesalpinia ferrea

Caesalpinia pulcherrima

Caesalpinia sappan

Cajanus cajan

Calendula officinalis

Callyspongia siphonella

Calophyllum brasiliense

Calophyllum inophyllum

Calotropis procera

Calycopteris floribunda

Camellia sinensis

Cananga

Cannabis sativa

Euphorbia lathyris

Capparis spinosa

Capsicum

Caralluma tuberculata

Carex folliculata

Careya arborea

Carica papaya

Carissa spinarum

Carpesium abrotanoides

Carthamus tinctorius

Casearia membranacea

Casearia sylvestris

Casimiroa edulis

Cassia alata

Cassia auriculata

Cassia fistula

Cassia occidentalis

Cassia tora

Cassipourea lanceolata

Catha edulis

Cedrus deodara

Celastrus orbiculatus

Celastrus rosthornianus

Centaurea arenaria

Centaurea deflexa

Cephalaria gigantea

Cephalotaxus harringtonia

Ceratodictyon spongiosum

Ceratodon purpureus

Ceratonia siliqua L.

Cerbera manghas

Cerbera odollam

Certonardoa semiregularis

Cespitularia

Cestrum nocturnum

Cetraria aculeata

Chaenomeles japonica

Chaetomium cochliodes

Chaetomium globosum

Cheilanthes farinosa

Chelidonium majus

Chenopodium album

Chenopodium ambrosioides

Cichorium intybus

Chisocheton erythrocarpus

Chisocheton siamensis

Chlamys farreri

Chloranthus henryi

Chloranthus japonicus

Chlorella vulgaris

Chlorophytum borivilianum

Chlorophytum orchidastrum

Chondrostereum

Chondrus ocellatus

Chromobacterium violaceum

Chromolaena odorata

Chrysanthemum coronarium

Chrysanthemum indicum

Chrysanthemum morifolium

Chrysopogon aciculatus

Chrysosporium

Chrysothamnus viscidiflorus

Chukrasia tabularis

Cibotium barometz

Cimicifuga dahurica

Cimicifuga foetida

Cimicifuga heracleifolia

cimicifuga racemosa

Cimicifuga yunnanensis

Cinachyrella

Cinchona officinalis

Cinnamomum cassia

Cinnamomum kotoense

Cinnamomum osmophloeum

Cinnamomum subavenium

Cinnamomum tenuifolium

Cinnamomum zeylanicum

Cirsium japonicum

Cistanche deserticola

Cistanche salsa

Citrullus colocynthis

Citrus aurantifolia

Citrus limon

Citrus natsudaidai

Citrus reticulata

Citrus sinensis

Citrus unshiu

Cladiella australis

Cladobotryum

Cladonia furcata

Clausena lansium

Clavularia koellikeri

Clavularia viridis

Celastrus kusanoi

Cleistanthus collinus

Clematis ganpiniana

Cleome gynandra

Clerodendron inerme

Clerodendrum cyrtophyllum

Cliona varians

Clitocybe alexandri

Clitocybe nebularis

Clusia paralicola

Cnidium japonicum

Cnidium monnieri

Cocculus orbiculatus

Codium fragile

Codonopsis lanceolata

Cordyceps cicadae

Coffea arabica

Coix lachryma-jobi

Colchicum crocifolium

Colchicum tunicatum

Coleus xanthanthus

Colocasia esculenta

Comanthus parvicirrus

Commiphora myrrha

Comptonia peregrina

Concholepas concholepas

Coprinus cinereus

Coprinus comatus

coprinus disseminatus

Coptis chinensis

Coptis groenlandica

Coptis japonica

Corchorus olitorius

Cordyceps bassiana

Cordyceps militaris

Cordyceps sinensis

Dupuya madagascariensis

Coriolus Versicolor

Cornus officinalis

Securigera varia

Corticium

Corydalis heterocarpa

Corydalis yanhusuo

Corynespora

Crambe crambe

Crassostrea gigas

Crassostrea virginica

Crataegus pinnatifida

Cratoxylum cochinchinense

Cribrochalina vasculum

Crinum asiaticum

Crocus boryi

Crocus niveus

Crocus sativus

Crotalaria agatiflora

Crotalaria sessiliflora

Croton cajucara

Croton insularis

Croton malambo

Cryptomeria japonica

Cucumaria frondosa

Cucurbita andreana

Cudrania tricuspidata

Culcita novaeguineae

Cuminum cyminum

Cupressus lusitanica

Cupressus sempervirens

Curculigo orchioides

Curcuma longa

Curcuma parviflora

Curcuma wenyujin

Curcuma xanthorrhiza

Curcuma zedoaria

Cuscuta chinensis

Cuscuta reflexa Roxb.

Cyathostemma argenteum

Cydonia oblonga

Cymbopogon flexuosus

Cynanchum auriculatum

Cynanchum vincetoxicum

Cynanchum wilfordii

Cynara cardunculus

Cynara scolymus

Cynodon dactylon

cynometra ramiflora

Cyperus alopecuroides

Cyperus rotundus

Cyprinus carpio

Cystodytes dellechiajei

Cystoseira myrica

Dactylospongia elegans

Trametes gibbosa

Dalbergia parviflora

Daldinia concentrica

Daphne genkwa

Daphne gnidium

Daphne tangutica

Datura inoxia

Datura metel

Datura stramonium

Delphinium chrysotrichum

Dendranthema morifolium

Dendrilla nigra

Dendrobium chrysotoxum

Dendrobium loddigesii

Dendropanax arboreus

Dendrophyllia cornigera

Deprea subtriflora

Dercitus bucklandi

Dermatophagoides farinae

Derris trifoliata

Dianthus caryophyllus

Dianthus superbus

Dichapetalum gelonioides

Dichroa febrifuga

Dictamnus dasycarpus

Digitalis purpurea

Dillenia indica

Dimocarpus longan

Dionysia termeana

Dioon spinulosum

Dioscorea collettii

Diospyros kaki

Diospyros lotus

Diospyros montana

Diospyros montana Roxb.

Diospyros virginiana

Diplazium esculentum

Diplosoma virens

Dipsacus asper

Discodermia dissoluta

Dodonaea viscosa

Dolabella auricularia

Dolichos

Draba nemorosa

Dracaena angustifolia

Dracaena arborea

Dracocephalum kotschyi

Drynaria fortunei

Dryopteris crassirhizoma

Duguetia hadrantha

Dunaliella salina

Dunaliella tertiolecta

Dysidea avara

Dysidea fragilis

Dysosma versipellis

Echinacea angustifolia

Echinacea pallida

Echinacea purpurea

Echinocystis lobata

Echinops bannaticus

Ecklonia cava

Eclipta prostrata

Ecteinascidia turbinata

Elaeagnus glabra

Elaeagnus umbellata

Elaeocarpus chinensis

Elaeocarpus hainanensis

Elaeodendron

Elephantopus mollis

Elephantopus scaber

Elettaria cardamomum

Eleutherine

Eleutherococcus senticosus

Emblica officinalis

Emericella falconensis

Emericella rugulosa

Emericella variecolor

Entada rheedii

Enterolobium contortisiliquum

Enteromorpha intestinalis

Ephedra sinica

Epilobium angustifolium

Epimedium koreanum

Epimedium sagittatum

Equisetum arvense L.

Eriobotrya japonica

Eruca sativa

Erythrina abyssinica

Erythropodium caribaeorum

Erythroxylum rotundifolium

Eubacterium lentum

Eubacterium rectale

Eucalyptus cypellocarpa

Eucalyptus grandis

Eucalyptus occidentalis

Eucomis autumnalis

Eucommia ulmoides

Eudendrium racemosum

Eudistoma

Eugenia caryophyllata

Eugenia jambolana

Euglena gracilis

Eulophia nuda

Eulophia petersii

Eunicea

Euonymus alatus

Euonymus sieboldianus

Eupatorium adenophorum

Eupatorium odoratum

Eupenicillium

Euphausia

Euphorbia ebracteolata Hayata

Euphorbia fischeriana

Euphorbia jolkini

Euphorbia kansui

Euphorbia lagascae

Euphorbia lathyris

Euphorbia pekinensis

Euphorbia peplus

Euphorbia socotrana

Euphorbia wallichii

Euplotes crassus

Eupolyphaga sinensis

Euphorbia helioscopia

Eurya emarginata

Eurycoma longifolia

Eurycoma longifolia Jack

Euscaphis japonica

Evernia prunastri

Evodia rutaecarpa

Exophiala dermatitidis

Fagonia boveana

Fagopyrum esculentum

Fagopyrum tataricum

Fagus sylvatica

Farfugium japonicum

Fascaplysinopsis

Ferula badrakema

Ferula communis

Ferula diversivittata

Ferula elaeochytris

Ferula kuhistanica

Ferula szowitsiana

Ferula vesceritensis

Ferulago angulata

Ferulago campestris

Fibraurea recisa

Fibraurea tinctoria

Ficus awkeotsang Makino

Ficus bengalensis

Ficus carica

Ficus deltoidea

Ficus formosana

Ficus hispida

Ficus microcarpa

Ficus pumila

Ficus racemosa

Ficus religiosa

Ficus septica

Filipendula ulmaria

Flammulina velutipes

Flavobacterium uliginosum

Flavoparmelia caperata

Flemingia philippinensis

Flemingia strobilifera

Flueggea virosa

Flustra foliacea

Foeniculum vulgare

Fomes fomentarius

Forcepia

Fragaria ananassa

Frangula alnus

Fraxinus sieboldiana

Fritillaria ussuriensis

Arctium minus

Fuligo candida

Fumaria

Fusarium equiseti

Gaillardia aristata Pursh

Galanthus alpinus

Galanthus krasnovii

Galanthus lagodechianus

Galanthus platyphyllus

Galanthus woronowii

Galanthus nivalis

Galaxaura marginata

Galium verum L.

Galphimia glauca

Gambierdiscus

Ganoderma colossum

Ganoderma lucidum

Ganoderma tsugae

Garcinia cowa

Garcinia hanburyi

Garcinia indica

Garcinia mangostana

Gardenia jasminoides

Gardenia obtusifolia

Gardenia sootepensis

Gardneria ovata

Garuga pinnata

Gastrodia elata Blume

Gaultheria itoana Hayata

Geigeria alata

Geissospermum

Gelsemium elegans

Gelsemium sempervirens

Genista ephedroides

Genista sessilifolia

Genista tinctoria

Gentiana aristata

Geodia corticostylifera

Geodia cydonium

Geodia mesotriaena

Geranium thunbergii

Gersemia fruticosa

Geum japonicum

Geum quellyon

Gibberella fujikuroi

Gingko biloba

Ginkgo biloba

Glechoma hederacea

Gleditsia sinensis

Gliocladium

Glochidion eriocarpum

Glochidion zeylanicum

Gloiopeltis furcata

Gloriosa superba

Glossodoris atromarginata

Gluconacetobacter xylinus

Glycine max

Glycyrrhiza glabra

Glycyrrhiza uralensis

Gmelina asiatica

Gnidia kraussiana

Gomphrena macrocephala

Goniothalamus amuyon

Goniothalamus cheliensis

goniothalamus gardneri

Goniothalamus laoticus

Goniothalamus undulatus

Grifola frondosa

Guaiacum

Guignardia

Gymnascella dankaliensis

gymnema montanum

Gymnodinium breve

Gynostemma pentaphyllum

Gypsophila oldhamiana

Haematococcus pluvialis

Hagenia abyssinica

Halichondria okadai

Haliclona nigra

Halimeda discoidea

Halocynthia roretzi

Halorosellinia

Hamamelis virginiana

Hamelia patens

Hammada scoparia

Hannoa chlorantha

Haplophyllum

Harpagophytum procumbens

Callichilia barteri

Hedychium coronarium

Hedychium gardnerianum

Hedychium spicatum

Hedyotis corymbosa

Hedyotis diffusa

Helianthus tuberosus

Helicteres angustifolia

Helicteres hirsuta

Hemerocallis fulva

Hemidesmus indicus

Hemsleya amabilis

Heracleum lanatum

Heracleum sibiricum

Hericium erinaceus

Hernandia nymphaeifolia

Hernandia peltata

Hibiscus sabdariffa

Hibiscus syriacus

Hibiscus vitifolius

Himatanthus drasticus

Hippophae rhamnoides

Hippospongia metachromia

Hippospongia

Holarrhena curtisii

Holothuria leucospilota

Homophymia

Hordeum vulgare

Humulus lupulus

Hunteria zeylanica

Hydrastis canadensis

Hydrocotyle bonariensis

Hydrocotyle sibthorpioides

Hymenaea courbaril

Hymeniacidon

Hymenocallis littoralis

Hypericum annulatum

Hypericum ascyron

Hypericum empetrifolium

Hypericum erectum

Hypericum hookerianum

Hypericum mysorense

Hypericum patulum

Hypericum perforatum

Hypericum rumeliacum

Hypericum sampsonii

Hypericum triquetrifolium

Hypocrea

Hypogymnia physodes

Hyrtios erecta

Imperata cylindrica

Inocybe umbrinella

Inonotus obliquus

Inula britannica

Inula cappa

Inula helenium

Inula japonica

Inula racemosa

Inula viscosa

Iotrochota

Ipomoea arborescens

Ipomoea batatas

Ipomoea murucoides

Ipomoea obscura

Ipomoea squamosa

Ipomoea stans

Ircinia ramosa

Iris tectorum

Irpex lacteus

Iryanthera lancifolia

Isaria tenuipes

Isatis indigotica

Isis hippuris

Isodon eriocalyx

Isodon melissoides

Isodon rubescens

Isodon sculponeatus

Ixeris

Ixora coccinea

Jaborosa cabrerae

Jaborosa reflexa

Quassia amara

Janthinobacterium

Jasminum grandiflorum

Jaspis splendens

Jatropha curcas

Jatropha multifida

Juglans mandshurica

Juglans regia

Junceella fragilis

Junceella juncea

Juniperus brevifolia

Juniperus chinensis

Juniperus communis

Juniperus excelsa

Juniperus virginiana

Justicia patentiflora

Justicia procumbens

Kadsura ananosma

Kadsura longipedunculata

Kageneckia oblonga

Kalopanax pictus

Kandelia candel

Khaya senegalensis

Kielmeyera coriacea

Kirkpatrickia variolosa

Klyxum simplex

Knightia excelsa

Kniphofia foliosa

Kochia scoparia

Lactarius subvellereus

Lactobacillus acidophilus

Lactobacillus casei

Lactobacillus plantarum

Lactobacillus rhamnosus

Laggera alata

Lamellaria

Laminaria japonica

Lantana camara

Larrea divaricata

Lasiodiplodia theobromae

Laurencia intricata

Laurus nobilis

Lentinus edodes

Leonurus heterophyllus

Leonurus japonicus

Lepidium apetalum

Lepidium sativum

Leptadenia reticulata

Leptogorgia sarmentosa

Lethariella

Leucetta chagosensis

Leucetta microraphis

Leucojum aestivum

Leuconotis

Leucopaxillus giganteus

Ligularia fischeri

Ligularia lapathifolia

Ligularia nelumbifolia

Ligularia platyglossa

Limnophila aromatica

Lindera strychnifolia

Linum flavum

Linum usitatissimum

Liquidambar formosana

Lissoclinum bistratum

Lissoclinum patella

Lissoclinum badium

Litchi chinensis

Lithospermum erythrorhizon

Lithothamnion

Litsea acutivena

Livistona chinensis

Lobelia inflata

Lobophora variegata

Lobophytum crassum

Lonchocarpus

Lonicera japonica

Lophocladia

Luffa aegyptiaca

Luffa cylindrica

Luffariella geometrica

lycium barbarum

Lycopodium clavatum

Lyngbya bouillonii

Lyngbya polychroa

Lyngbya majuscula

Mahonia oiwakensis

Marrubium vulgare

Marsdenia tenacissima

Matricaria chamomilla

Matricaria recutita

Melaleuca Alternifolia

Melissa officinalis

Mentha arvensis

Mentha longifolia

Mentha piperita

Mentha spicata

Mentha pulegium

Microcystis aeruginosa

Microcystis?viridis

Micromonospora marina

Micromonospora chalcea

Micromonospora lupini

Microsphaeropsis

Momordica charantia

Momordica cochinchinensis

Montipora digitata

Morinda citrifolia

Morus alba

Musa paradisiaca

Musa sapientum

Mycale hentscheli

Mycale magellanica

Mylabris

Myrica rubra

Myristica fragrans

Narcissia

Nelumbo nucifera

Neosiphonia

Nephthea

Nocardiopsis

Nostoc sp.

Ocimum basilicum

Ocimum gratissimum

Ocimum sanctum

Ocimum viride

Oenothera biennis

Oldenlandia diffusa

Olea europaea

Oncorhynchus keta

Opuntia ficus-indica

opuntia

Origanum dictamnus

Origanum majorana

Oryza sativa

Pachyclavularia

Paeonia lactiflora

panax ginseng

Panax notoginseng

Panax quinquefolius

Penicillium

Pentadesma butyracea

Peperomia pellucida

Peperomia tetraphylla

Perezia

Pergularia tomentosa

Periconia byssoides

Perilla frutescens

Periplaneta Americana

Periploca graeca

Peronema canescens

Perovskia abrotanoides

Persea americana

Persea obovatifolia

Pestalotiopsis fici

Petalonia binghamiae

Petasites formosanus

Petiveria alliacea

Petroselinum crispum

Petrosia

Peucedanum japonicum

Peucedanum nebrodense

Peucedanum praeruptorum

Peumus boldus

Xanthophyllomyces dendrorhous

Phakellia fusca

Phalaris canariensis

Phallusia nigra

Pharbitis nil

Phaseolus acutifolius

Phaseolus angularis

Phaseolus coccineus

Phaseolus vulgaris

Phellinus linteus

Phellodendron amurense

Phialophora gregata

Philinopsis speciosa

Pholidota yunnanensis

Pholiota adiposa

Pholiota spumosa

Phomopsis leptostromiformis

Phomopsis longicolla

Phomopsis asparagi

Phormidium tenue

Phyllanthus amarus

Phyllanthus emblica

Phyllanthus polyphyllus

Phyllanthus pulcher

Phyllanthus virgatus

Phyllostachys edulis

Phyllostachys nigra

Phyllosticta spinarum

Physalis angulata L.

Physalis longifolia

Physalis minima L.

Physalis peruviana

Physarum polycephalum

Phytolacca acinosa

Phytolacca americana

Phytophthora

Picea abies

Picramnia latifolia

Picrasma quassioides

Picrolemma sprucei

Picrorhiza kurroa

Pieris brassicae

Pieris melete

Pieris rapae

Pimpinella anisum

Pimpinella corymbosa

Pinellia pedatisecta Schott

Pinus densiflora

Pinus massoniana

Pinus massoniana

Pinus parviflora

Pinus yunnanensis

Piper betle

Piper cubeba

Piper kadsura

Piper longum

Piper methysticum

Piper Nigrum

Piptadenia adiantoides

Pisolithus tinctorius

Pistacia lentiscus

Pisum sativum

Pityrogramma calomelanos

Plakinastrella onkodes

Plakortis

Planaxis sulcatus

Plantago afra

Plantago asiatica

Plantago bellardii

Plantago lagopus

Plantago lanceolata

Plantago major

Plantago serraria

plasmodium falciparum

Platismatia glauca

Platycladus orientalis

Platycodon grandiflorum

Platymiscium floribundum

Pleurobranchus forskalii

Pleurotus abalonus

Pleurotus citrinopileatus

Pleurotus eryngii

Pleurotus nebrodensis

Pleurotus ostreatus

Pleurotus pulmonarius

Pleurotus sajor-caju

Pleurotus tuber-regium

Pluchea odorata

Plumbago zeylanica

Podocarpus macrophyllus

Podocarpus madagascariensis

Podophyllum hexandrum

Podoscypha

Poecillastra

Pogonopus speciosus

Polyalthia longifolia

Polyalthia rumphii

Polycarpa aurata

Gracilaria edulis

Polygala senega

Polygonatum cyrtonema

Polygonatum odoratum

polygonum aviculare

Polygonum Cuspidatum

Polygonum limbatum

polygonum multiflorum

Polygonum tinctorium

Polymastia janeirensis

Polymastia tenax

Polyporus umbellatus

Polysiphonia japonica

Polysiphonia lanosa

Polytrichum commune

Polytrichum pallidisetum

Poncirus trifoliata

Pongamia pinnata

Poria cocos

Porphyra yezoensis

Porphyridium cruentum

Portulaca oleracea

Potamogeton crispus

Potentilla argentea

Potentilla erecta

Premna

Primula denticulata

Primula obconica

Prorocentrum mexicanum

Prosopis cineraria

Prunella vulgaris

Prunus africana

Prunus amygdalus

Prunus armeniaca

Prunus cerasus

Prunus Domestica

Prunus dulcis

Prunus mume

Prunus persica

Prunus salicina

Prunus serotina

Prunus serrulata

Prunus yedoensis

Prunus zippeliana

Prymnesium parvum

Psammaplysilla purpurea

Psammocinia

Psathyrella

Pseudevernia intensa

Pseudobersama mossambicensis

Pseudolarix kaempferi

Pseudomonas sp

Pseudoplexaura wagenaari

Pseudopterogorgia acerosa

Psidium cattleianum Sabine

Psidium guajava

Psilaster cassiope

Psorothamnus emoryi

Psychotria horizontalis

Psychotria leptothyrsa

Pteris semipinnata

Pteris multifida

Pterocarpus marsupium

Pterocarpus santalinus

Pterocarya stenoptera

Pterocaulon polystachyum

Pterodon pubescens

Pterodon pubescens

Pterospermum heterophyllum

Ptilota filicina

Karenia brevis

Pueraria mirifica

Pueraria thomsonii

Pulsatilla chinensis

Pulsatilla koreana

Punica granatum

Punica protopunica

Prunus africana

Pyrularia pubera

Quassia africana

Quercus robur

Quercus suber

Quillaja saponaria

Quisqualis indica

Rabdosia coetsa

Rabdosia japonica

Rabdosia rubescens

Radianthus macrodactylus

Ramalina celastri

Rana catesbeiana

Raphanus sativus

Raspailia

Ratibida columnifera

Rauwolfia serpentina

Reaumuria vermiculata

Rehmannia glutinosa

Reissantia buchananii

Reniera fulva

Reticulitermes speratus

Rhamnus alaternus

Rhamnus purshiana

Rhaponticum carthamoides

Rheum emodi

Rheum officinale

Rheum palmatum

Rheum rhaponticum

Rheum ribes

Rheum undulatum

Rhinacanthus nasutus

Rhizobium

Rhizophora apiculata

Rhizophora mucronata

Rhizopus arrhizus

Rhizopus oryzae

Rhodiola algida

Rhodiola crenulata

Rhodiola sachalinensis

Rhododendron ferrugineum

Rhodomela confervoides

Rhodopseudomonas palustris

Rheum rhabarbarum

Rhus pyroides

Rhus succedanea

Rhynchosia volubilis

Ribes rubrum

Ricinus communis

Robinia pseudoacacia

Rolandra fruticosa

Roldana angulifolia

Rollinia mucosa

Rosa rugosa

Rosmarinus officinalis

Roupellina boivinii

Rozites

Rubia cordifolia

Rubia peregrina

Rubia tinctorum

Rubia yunnanensis

Rubus chamaemorus

Rubus coreanus

Rubus crataegifolius

Rubus idaeus

Rubus occidentalis

Rubus parvifolius

Rumex acetosella

Rumex dentatus

Rumex maritimus

Russula clelandii

Russula cyanoxantha

Russula delica

Ruta graveolens

Saccharopolyspora

Saccharothrix aerocolonigenes

Saccharum officinarum

Salacia oblonga

Salicornia herbacea

Salix alba

Salix caprea

Salix purpurea

Salix safsaf

Salpichroa origanifolia

Salsola oppositifolia Desf.

Salvadora persica

Salvia austriaca

Salvia castanea

Salvia chinensis

Salvia coccinia

Salvia corrugata

Salvia digitaloides

Salvia dominica

Salvia fruticosa

Salvia officinalis

Salvia sclarea

Salvia spinosa

Sambucus nigra

Sandoricum koetjape

Sanguinaria canadensis

Sanguisorba minor

Sanguisorba officinalis

Sanicula lamelligera

Sansevieria ehrenbergii

Santalum album

Saponaria officinalis

Saponaria vaccaria

Sarcomelicope

Sarcophyton ehrenbergi

Sarcophyton elegans

Sarcophyton glaucum

Sarcotragus

Sargassum carpophyllum

Sargassum filipendula

Sargassum fusiforme

Sargassum vachellianum

Sargassum latifolium

Satureja hortensis

satureja montana

Sauromatum venosum

Saururus chinensis

Saussurea costus

Saussurea involucrata

Saussurea lappa

Saussurea salicifolia

Schefflera heptaphylla

Schinus Molle

Schinus Terebinthifolius

Schisandra chinensis

Schisandra grandiflora

Schisandra propinqua

Schizophyllum commune

Scilla scilloides

Sclerotium rolfsii

Scoparia dulcis

Scorzonera austriaca

Scrophularia ningpoensis

Scutellaria baicalensis

Scutellaria barbata

Scutellaria orientalis

Securinega suffruticosa

Sedum sarmentosum

Selaginella doederleinii

Selaginella moellendorffii

Selaginella willdenowii

Selaginella tamariscina

Senecio latifolius

sepiella maindroni

serenoa repens

Serratia marcescens

sesamum indicum

Sesbania grandiflora

Seseli annuum L.

Setipinna taty

Shorea gibbosa

Shorea roxburghii

Sideritis trojana

Silybum marianum

Simaba cedron

Simarouba glauca

Sinularia capillosa

Sinularia dura

Sinularia flexibilis

Sinularia gibberosa

Sinularia grandilobata

Sinularia granosa

Sinularia inelegans

Sinularia manaarensis

Sinularia maxima

Sinularia nanolobata

Sinularia polydactyla

Sinularia scabra

Skeletonema costatum

Smallanthus sonchifolius

Smilax china

Smilax domingensis

Smilax myosotiflora

Solanum incanum

Solanum lyratum

Solanum nigrum L.

Solanum torvum

Solanum violaceum

Sophora flavescens

Sorangium cellulosum

Sorghum bicolor

Soymida febrifuga

Sparassis crispa

Sparganium stoloniferum

Spatholobus suberectus

Sphaerococcus coronopifolius

Spicaria elegans

Spirastrella

Spirulina maxima

Spirulina platensis

Spongosorites

Stachys officinalis

Stachys byzantina

Stachys germanica

Stachys palustris

Stachys recta

Stephania japonica

Steganotaenia araliacea

Stelletta

Stemona aphylla

Stemona curtisii

Stemona tuberosa

Stephania cepharantha

Stephania rotunda

Stephania tetrandra

stevia rebaudiana

Streblus asper

Streptocarpus dunnii

Streptomyces candidus var. azaticus

Streptomyces spiroverticillatus

Strobilanthes crispus

Strobilurus tenacellus

Strongylocentrotus intermedius

Strongylocentrotus nudus

Strychnos icaja

Strychnos nuxvomica

Styela plicata

Stypopodium zonale

Stypopodium flabelliforme

Subergorgia suberosa

Suillus granulatus

Suillus luteus

Suillus placidus

Swertia chirata

Swertia punicea

Symphonia

Symplocos chinensis

Syzygium siamense

Tabanus

Handroanthus impetiginosus

Tabernaemontana calcarea

Tabernaemontana divaricata

Tabernaemontana sphaerocarpa

Tacca chantrieri

Tacca subflabellata

Talaromyces flavus

Talaromyces wortmannii

Tamarindus indica

Tambourissa

Tanacetum parthenium

Tannerella forsythia

Taraxacum mongolicum

Taraxacum officinale

Taxus chinensis

Taxus chinensis var. mairei

Taxus cuspidata

Taxus cuspidata var. nana

Taxus mairei

Taxus wallichiana

Taxus yunnanensis

Tecomella undulata

Telesto

Tephrosia calophylla

Tephrosia purpurea

Terminalia arjuna

Terminalia calamansanai

Terminalia catappa

Terminalia chebula

Terminalia ivorensis

Teucrium polium

Thalictrum acutifolium

Thelephora aurantiotincta

Theobroma cacao

Theobroma grandiflorum

Theonella sp.

Theonella swinhoei

Thespesia populnea

Cascabela thevetia

Thorectandra

Thuja occidentalis

Thujopsis dolabrata

Tiliacora

Tinospora cordifolia

Tithonia diversifolia

Toona sinensis

Tovomita longifolia

Trichoderma atroviride

Trichoderma harzianum

Trichoderma virens

Tricholoma equestre

Trichosanthes anguina

Trichosanthes kirilowii

Trididemnum solidum

Trifolium pratense

Trigona laeviceps

Trigonella foenum-graecum

Phyllanthus emblica

Tripterygium hypoglaucum

Tripterygium regelii

Tripterygium wilfordii

Triticum aestivum

Tupistra chinensis

Turbinaria conoides

Turraea pubescens

Tussilago farfara L.

Tydemania expeditionis

Tylophora tanakae

Udotea flabellum

Ulmus davidiana

Ulmus davidiana var. japonica

Ulmus laevis

Ulmus pumila

Umbilicaria crustulosa

Umbilicaria cylindrica

Umbilicaria esculenta

Umbilicaria polyphylla

Uncaria rhynchophylla

Uncaria tomentosa

Undaria pinnatifida

Urtica dioica

Uvaria

Vaccinium macrocarpon

Vaccinium myrtillus

Valeriana jatamansi

Valeriana sorbifolia

Ventilago madraspatana Gaertn.

Veratrum dahuricum

Verbascum thapsus

Vernonia cinerea

Veronica cymbalaria

Veronica hederifolia

Veronica pectinata

Veronica persica

Veronica polita

Verrucosispora

Vespa simillima

Viburnum awabuki

Viburnum luzonicum

Vigna angularis

Vigna unguiculata

Viola arvensis

Viola odorata

Virgularia juncea

Viscum album

Vismia baccifera

Vismia macrophylla

Vitex agnus-castus

Vitex negundo

Vitex rotundifolia

Vitex trifolia

Vitis vinifera

Dolomiaea calophylla

Wasabia japonica

Wilbrandia ebracteata

Withania somnifera

Xanthoceras sorbifolia

Xestospongia exigua

Xestospongia exigua

Ximenia americana

Xylocarpus granatum

Xylopia aethiopica

Xylopia aromatica

Youngia japonica

Yucca schidigera

Zanthoxylum ailanthoides

Zanthoxylum americanum

Zanthoxylum leprieurii

Zanthoxylum piperitum

Zanthoxylum schinifolium

Zea mays

Zingiber officinale

Zinnia grandiflora

Ziziphus mauritiana

Zizyphus jujuba

Zuccagnia punctata

Zygogynum

Zyzzya fuliginosa
